# Speciation Controls the Kinetics of Iron Hydroxide Precipitation and Transformation at Alkaline pH

**DOI:** 10.1021/acs.est.4c06818

**Published:** 2024-10-23

**Authors:** Fabio E. Furcas, Shishir Mundra, Barbara Lothenbach, Ueli M. Angst

**Affiliations:** † Institute for Building Materials, ETH Zürich, Laura-Hezner-Weg 7, 8093 Zürich, Switzerland; ‡ Empa Concrete & Asphalt Laboratory, Ueberlandstrasse 129, 8600 Dübendorf, Switzerland

**Keywords:** precipitation, iron, kinetics, pH, partial equilibrium

## Abstract

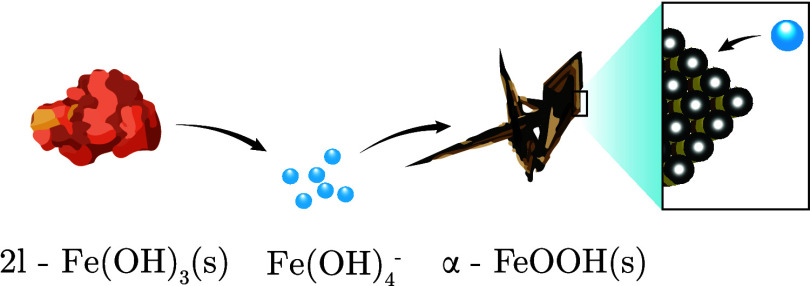

The formation of energetically favorable and metastable mineral phases within the Fe–H_2_O system controls the long-term mobility of iron complexes in natural aquifers and other environmentally and industrially relevant systems. The fundamental mechanism controlling the formation of these phases has remained enigmatic. We develop a general partial equilibrium model, leveraging recent synchrotron-based data on the time evolution of solid Fe(III) hydroxides along with aqueous complexes. We combine thermodynamic considerations and particle-morphology-dependent kinetic rate equations under full consideration of the aqueous phase in disequilibrium with one or more of the forming minerals. The new model predicts the rate of amorphous 2-line ferrihydrite precipitation, dissolution, and overall transformation to crystalline goethite. It is found that the precipitation of goethite (i) occurs from solution and (ii) is limited by the comparatively slow dissolution of the first forming amorphous phase 2-line ferrihydrite. A generalized transformation mechanism further illustrates that differences in the kinetics of Fe(III) precipitation are controlled by the coordination environment of the predominant Fe(III) hydrolysis product. The framework allows modeling of other iron(bearing) phases across a broad range of aqueous phase compositions.

## Abstract 2

The transformation of 2-line ferrihydrite is rate-limited by its dissolution as aqueous Fe(III) across a broad range of natural and industrially relevant aqueous systems.

## Introduction

Depending on the aqueous phase composition and a range of other physiochemical parameters including temperature and pH, iron may precipitate in the form of over 38 stable and metastable phases characterized to date.^1^ Iron (hydr)oxides are the most common form of metallic oxides in soils.^2^ Their formation governs the immobilization of elements of concern (EOCs) including As, Se, Mo, Ni, and ^226^Ra in groundwater streams,^3^ soil environments,^4^ nuclear processing facilities,^5^ and across a broad range of other natural and industrial aqueous systems.^6−8^ Iron (hydr)oxide precipitation within the pore network of cementitious materials is one of the major causes of premature structural degradation of reinforced concrete structures.^9^ Iron uptake by calcium silicate hydrates (C–S–H) may further reduce the ability of cement-based nuclear waste repositories to contain and safely store hazardous radionuclides.^10,11^ Iron (hydr)oxides are also versatile industrial products used as pigments in the production of paints and coatings,^12^ in wastewater treatment,^13^ as well as in nanotechnology,^14^ photovoltaic,^15^ and energy storage systems.^16^ For these reasons, detailed knowledge about the mechanism and transformation kinetics of such iron (hydr)oxides is needed to assess their stability over different time scales and conditions.

**Figure 1:**
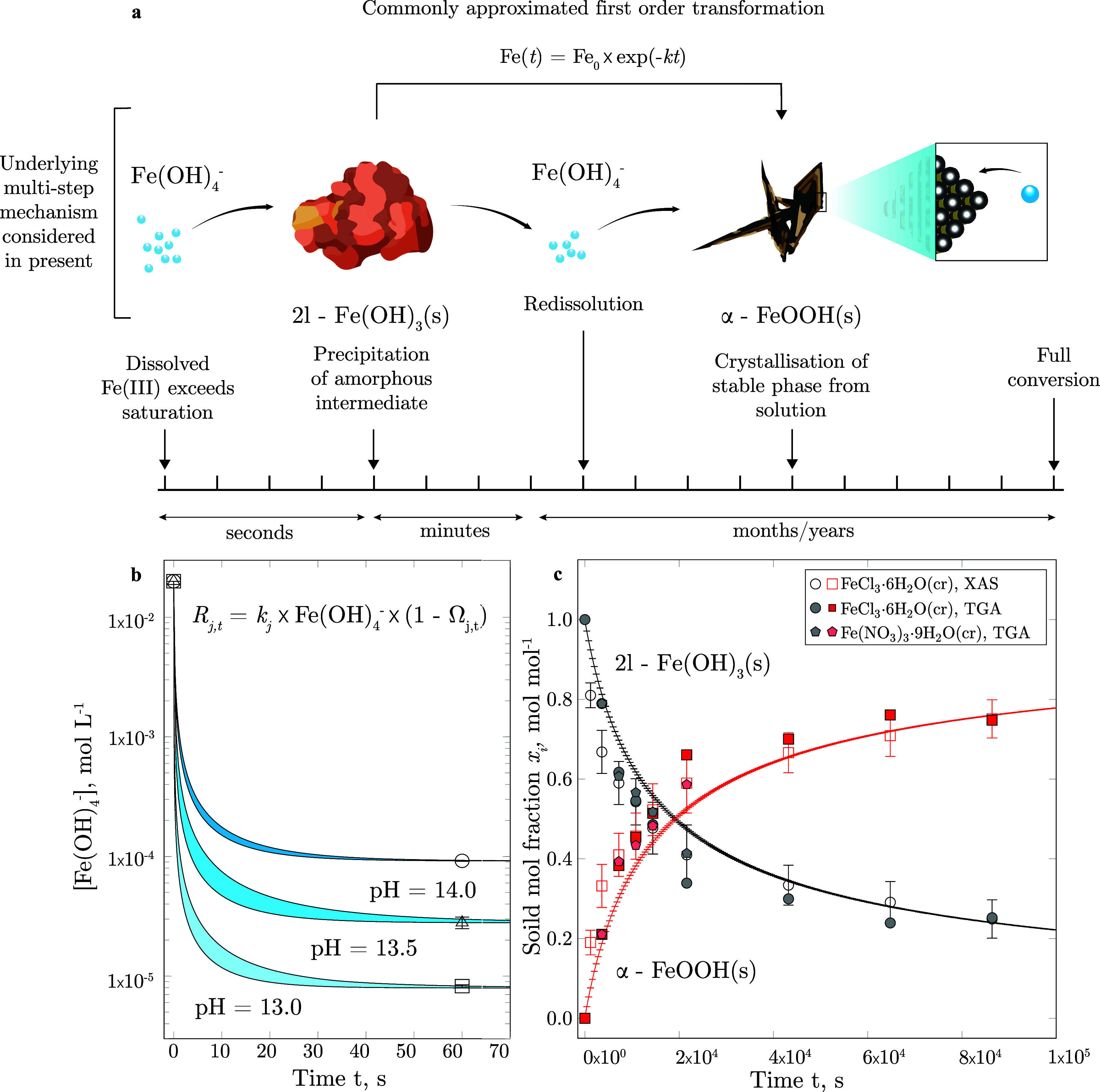
Schematic representation of the formation mechanism of crystalline iron (hydr)oxide phases from supersaturated aqueous solutions (a), together with the measured and computationally predicted aqueous iron concentration (b) and solid mole fractions (c). The initial precipitation of amorphous 2l–Fe(OH)_3_(s) from dissolved Fe(III) proceeds within seconds, full conversion to the stable end member α-FeOOH(s) is reached after months to years. This multistep conversion process is commonly approximated by the integrated first-order rate equation Fe(*t*) = Fe_0_ × exp(−*kt*), irrespective of the varying degree of supersaturation Ω and other aqueous phase parameters.

Investigations into the kinetics of iron (hydr)oxide precipitation primarily quantify the reaction rate, and thus its extent, by monitoring the molar fraction of solids formed, often assuming direct proportionality between their rate of formation and concentration.^17−20^ Two objections may be raised against this modeling approach. First, it is known that the formation and transformation of some iron (hydr)oxides proceeds via particle-mediated growth mechanisms,^21,22^ or involve metastable intermediate species^19^ (Figure 1a). As opposed to growth by the addition of singular atoms into an existing solid phase, as described within the framework of classical nucleation theory,^23^ these nonclassical growth mechanisms consist of multiple dissolution and precipitation steps and can thus not be described completely by the integrated first-order rate equation or any other semiempirical equation of the form Fe(*t*) = Fe_0_ × exp(*f*(*t*)). Second, mineral dissolution and growth rates are, among other parameters, dependent on the aqueous phase composition, the degree of supersaturation Ω, and the activity of dissolved species in disequilibrium with the solid phase(s). These parameters are generally not considered in first-order rate expressions. Moreover, due to the low solubility of iron, these parameters are significantly more difficult to obtain from an experimental point of view than the molar fraction of solid phases. In the simplest case, the irreversible formation of one ferrous (*z* = 2) or ferric (*z* = 3) iron (hydr)oxide according to the general reaction
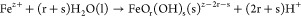
1is expected to depend on the Fe^*z*+^ and the H^+^ activity. In the context of natural and industrially relevant aqueous electrolytes, the phase assemblage of iron (hydr)oxides is significantly more complex. Consider the fate of Fe^2+^ due to the corrosion of carbon steel in near-neutral environments.

In the aqueous phase, the ferrous cation may be coordinated as FeOH^+^, Fe(OH)_2_(aq), or Fe(OH)_3_^–^, depending on the pH. These aqueous Fe(II) complexes may further oxidize, both aerobically and in the absence of oxygen, to form Fe^3+^ or any of the Fe(III) hydrolysis products FeOH^2+^, Fe(OH)_2_^+^, Fe(OH)_3_(aq), or Fe(OH)_4_^–^.^24^ The presence of carbonates, chlorides, silica, or any other anion characteristic to the aqueous environment of interest^5,25−27^ can lead to further complexation of the dissolved Fe(II) and Fe(III) hydrolysis products. The phase assemblage of solid iron(bearing) phases is thus in direct competition with the speciation of iron in the aqueous phase. From all of these considerations, it is evident that the mechanism fundamentally controlling the kinetics of iron (hydr)oxide formation can only be unraveled in a tightly coupled investigation of both the solid and the aqueous phase composition. Until recently, however, there was no data reported that comprehensively characterize the evolution of both the solid phases and the electrolyte composition at alkaline pH.

Recent studies^28,29^ reporting on the time evolution of solid Fe(III) hydroxides and complexes allow, for the first time, to model their formation mechanism in the Fe–H_2_O system under full consideration of the aqueous phase in disequilibrium with one or more of these minerals. On this basis, we develop a new partial equilibrium model, combining state-of-the-art thermodynamic parameters and particle-morphology-dependent kinetic rate equations. We use this model to demonstrate that the formation of goethite, a thermodynamically stable iron hydroxide, is controlled by the dissolution kinetics of amorphous 2-line ferrihydrite at alkaline pH. The proposed model simultaneously describes both the evolution of all solid and the aqueous phase constituents involved and breaks down the transformation mechanism into individual steps, thus rendering the need for various fitting parameters obsolete. All steps constituting the dissolution–crystallization pathway rely on a single kinetic rate constant. Upon considering the speciation of aqueous Fe(III) at neutral to mildly acidic pH, the transformation mechanism can be generalized to all thermodynamically stable solid Fe(III) phases, by adopting the Palandri–Kharaka kinetic formalism. Here, the overall rate of Fe(III) precipitation is limited by the intrinsic precipitation rate of the predominant hydrolyzed aqueous Fe(III) species, Fe(OH)_3_(aq) at circumneutral and Fe(OH)_4_^–^ at alkaline pH. These observations are in line with both Stranski’s rule^30^ and the Ostwald step rule.^31^ We envision this model to be expanded to a wider range of iron-bearing phases and aqueous systems.

## Results and Discussion

### The Precipitation of 2-Line Ferrihydrite at Alkaline pH

Recently, we have shown that the precipitation of 2-line ferrihydrite (2l–Fe(OH)_3_(s)) from supersaturated alkaline stock solutions (e.g., [Fe(III)] > 10^–4^ M at pH = 14.0) occurs significantly more rapidly than its transformation to more stable secondary phases including hematite (α-Fe_2_O_3_(s)) and goethite (α-FeOOH(s)).^28^ As over 99.8% of Fe(III) in solution is coordinated as Fe(OH)_4_^–^ at a pH ≥ 12,^32^ the precipitation of 2l–Fe(OH)_3_(s) at alkaline pH can be described by
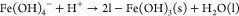
2Considering that phase growth velocity is anticipated to rise with increasing activity of Fe(OH)_4_^–^ and decline as saturation conditions are approached, we formulate the rate of 2-line ferrihydrite precipitation as
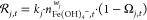
3for *j* = 2l–Fe(OH)_3_(s), ∀*t* (compare Supporting Information, List of symbols and notations). Correspondingly, the molar balance^1^ of all species *i* involved in the formation reaction displayed in eq 2 is
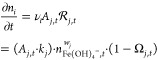
4To quantify the rate constant *k*_*j*_ and reaction order *w*_*j*_ as a function of the pH, the progression of [Fe(OH)_4_^–^] is fitted to the aqueous Fe(III) concentration, measured by inductively coupled plasma optical emission spectroscopy (ICP-OES) at pH = 13.0, 13.5, and 14.0, as published in Furcas et al.,^28^ within the first 60 s of equilibration time. It is assumed that the morphology of precipitated 2-line ferrihydrite does not change upon growth. Figure 1b displays the resultant concentration profiles at various pH values over time. It can be recognized that the precipitation rate drastically decreases, as the aqueous Fe(OH)_4_^–^ concentration approaches its pH-dependent solubility limit with respect to 2-line ferrihydrite. Within the pH interval investigated, the solubility limit of 2-line ferrihydrite increases by approximately 1 order of magnitude per pH unit. The pH dependence of  is therefore implicitly accounted for by the thermodynamic speciation solver. Within the error of the experimentally measured Fe(III) concentrations, the apparent rate constant of 2l–Fe(OH)_3_(s) precipitation is evaluated as (*k*_*j*_ · *A*_*j,t*_) = 0.078 ± 0.010 s^–1^, while the reaction order is *w*_*j*_ = 1 with respect to the Fe(OH)_4_^–^ concentration.

**Figure 2:**
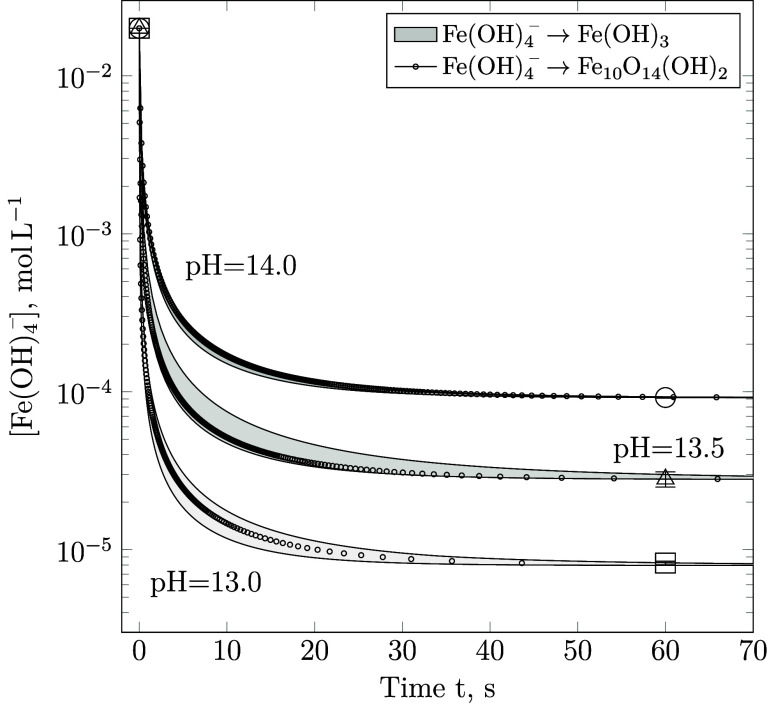
Comparison between the computationally predicted aqueous Fe(OH)_4_^–^ concentrations in mol/L, assuming their stoichiometric conversion to Fe(OH)_3_ (shaded regions), as given by eq 2 and to the ideal stoichiometric formula Fe_10_O_14_(OH)_2_ reported by Michel et al.^33^ (circular markers), shown in eq 5.

The here presented analysis also holds true for 2-line ferrihydrite stoichiometries other than the assumed Fe(OH)_3_(s). Assuming an ideal stoichiometry of Fe_10_O_14_(OH)_2_ in the absence of additional surface-bound water,^33^ the precipitation of 2-line ferrihydrite at alkaline pH proceeds according to
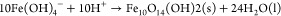
5While on a molar basis, the number of protons consumed in eqs 2 and 5 per equivalent of Fe(OH)_4_^–^ reacted off remains constant, it is evident that the ideal product stoichiometry reported in Michel et al.^33^ is significantly dryer than the assumed structural formula of Fe(OH)_3_, equivalent to the 2-line ferrihydrite standard reported in Furcas et al.^28^ As shown in Figure 2, the additional elimination of 1.4 equiv of H_2_O(l) per equivalent of iron does not compromise the goodness of the fitting results. In accordance with the 10-fold increase in the stoichiometric coefficient of Fe(OH)_4_^–^, the apparent rate constant of 2-line ferrihydrite precipitation decreases by a factor of 10, reaching values of (*k*_*j*_ · *A*_*j,t*_) = 0.008 ± 0.001 s^–1^. The reaction order remains proportional to the concentration of Fe(OH)_4_^–^.

### The Transformation of 2-Line Ferrihydrite to Goethite at Alkaline pH

**Figure 3:**
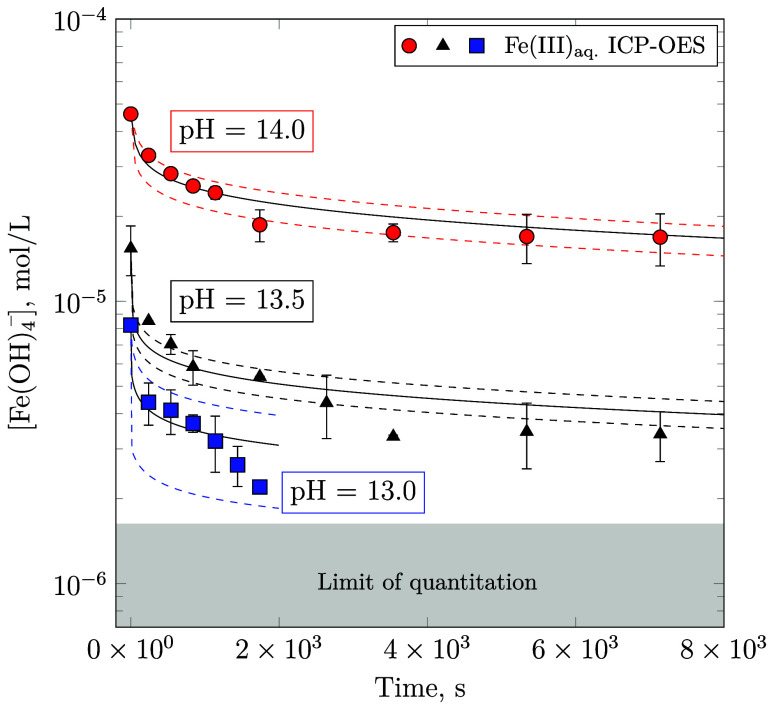
Combined modeling results (solid lines) considering the kinetics governing the rate of goethite formation (eq 9) and 2-line ferrihydrite dissolution (eq 10), matching the iron concentration measured by ICP-OES at pH = 14.0. Dashed lines represent the confidence intervals of the computationally predicted aqueous iron concentrations. Simulations correct for the specific surface area of both phases by applying a mass cubic root correction. Note that the computationally predicted solid molar fractions and aqueous iron concentrations are fitted to their experimental counterparts published in Furcas et al.^28^

For aqueous Fe(III) concentrations in-between the solubility limits of 2-line ferrihydrite and goethite (e.g., 10^–4^ > [Fe(III)] > 10^–7^ M at pH = 14.0), the aqueous Fe(OH)_4_^–^ concentration can increase due to the redissolution of 2l–Fe(OH)_3_(s) according to
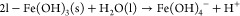
6and decrease due to the precipitation of goethite from solution
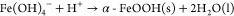
7Phase growth may also occur via aggregation-based mechanisms, involving the formation of iron–oxygen bonds due to the elimination of water
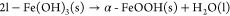
8Analogous to the formation of 2-line ferrihydrite (2l), the growth rate of goethite (gt) is expected to be primarily dependent on the activity of Fe(OH)_4_^–^ as well as the degree of supersaturation
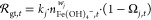
9for *j* = α-FeOOH(s), ∀*t*. In contrast, the rate of 2-line ferrihydrite dissolution
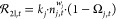
10for *j* = 2l–Fe(OH)_3_(s) and ∀*t* is found to be insensitive to the saturation index Ω, as the aqueous Fe(OH)_4_^–^ concentration remains close to the solubility limit of 2-line ferrihydrite (Supporting Information, Figure S1a). It is instead determined by the number of moles of *n*_2l,*t*_. The rate of aggregation-based growth of goethite from 2-line ferrihydrite does not involve the redissolution of Fe(OH)_4_^–^, and is thus written as
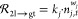
11*j* = 2l–Fe(OH)_3_(s), ∀*t*. Combining these rate expressions^2^, the molar species balances that describe the evolution of various species *i* involved in the dissolution (eq 6) and precipitation (eq 7) reaction are
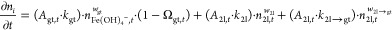
12Taking the initial specific surface area of 2-line ferrihydrite and goethite to be 6.0 × 10^5^ and 1.3 × 10^5^ m^2^ kg^–1^,^34^ the kinetic rate parameters of ∂*n*_*i*_/∂*t* are determined by fitting the predicted aqueous progression of [Fe(OH)_4_^–^] and the solid mole fraction *x*_*j*_ of both phases *j* ∈ Γ to the experimental data collected by Furcas et al.^28^ It is assumed that the specific surface area *A*_*s,j*_ scales with the phase mass in accordance with the cubic root correction formula displayed in eq 31. As illustrated in Figures 1 and 3, the calculated aqueous Fe(III) concentration and the solid phase assemblage are in good agreement with their experimental counterparts within the uncertainty associated with ICP-OES measurements and the estimated surface rate constants.

**Figure 4:**
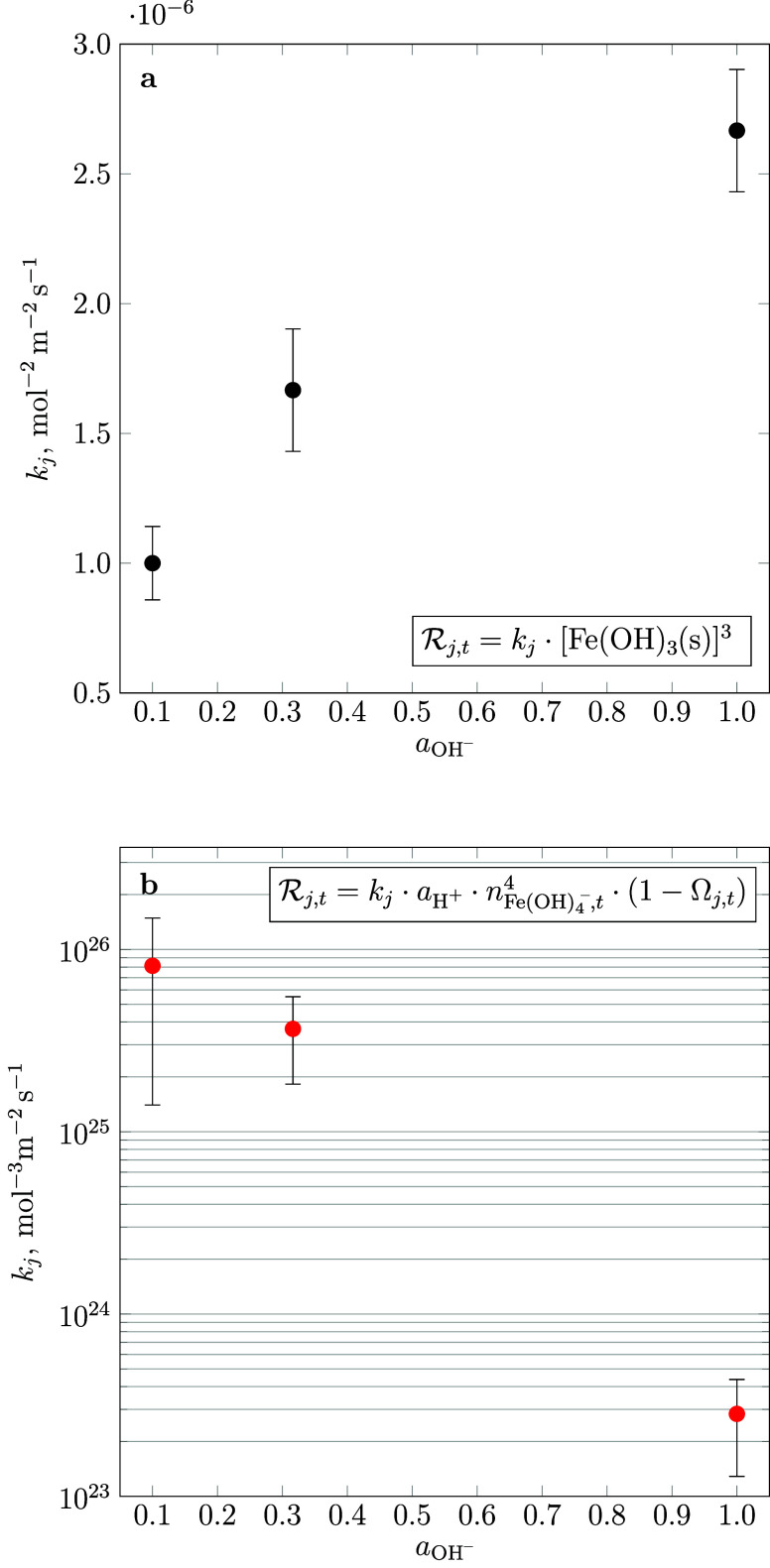
Surface rate constants *k*_*j*_ in mol^–3^ m^–2^ s^–1^ of two-line ferrihydrite dissolution (a) and goethite precipitation (b) as a function of pH.

**Figure 5:**
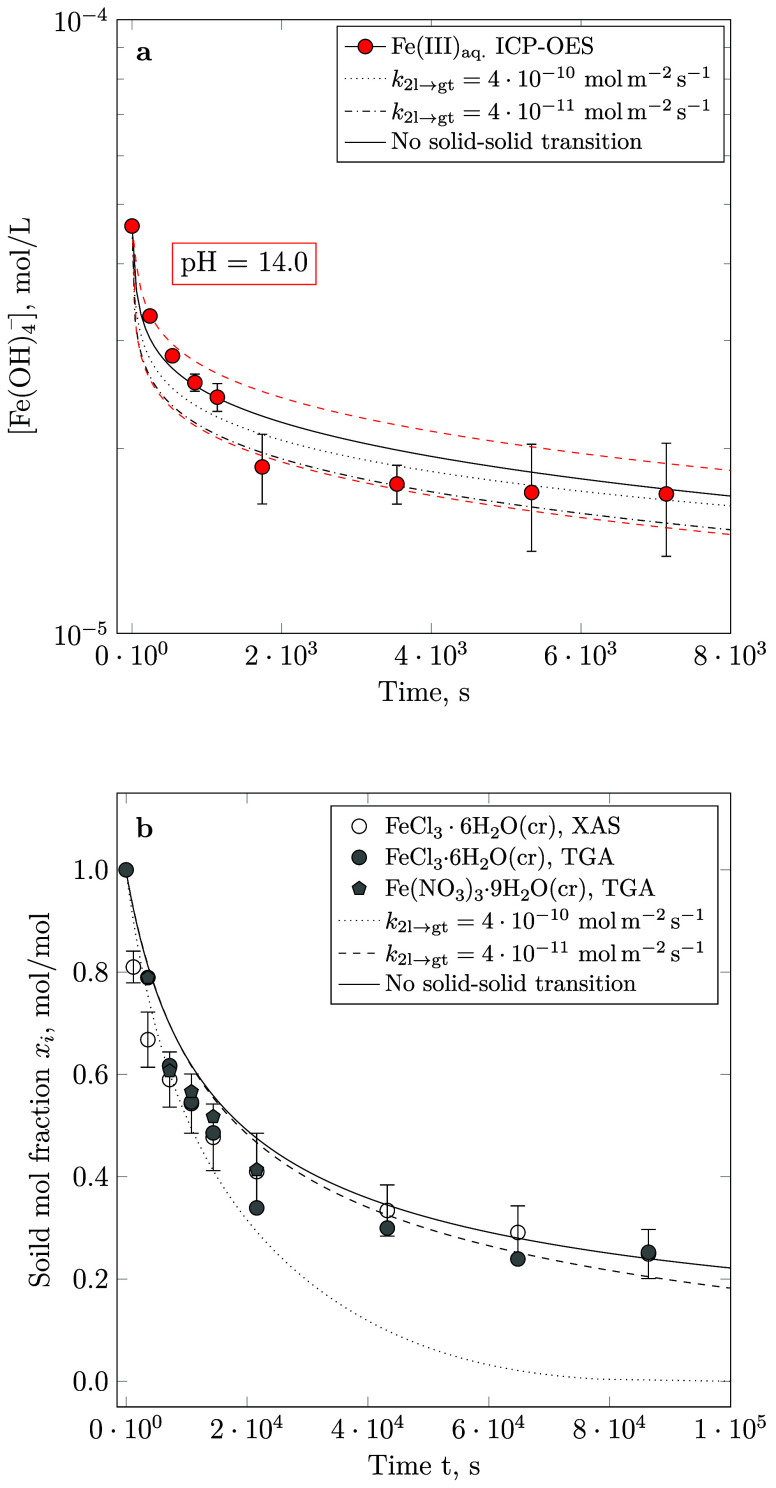
Predicted aqueous Fe(OH)_4_^–^ concentration (a) and solid molar fraction of 2-line ferrihydrite (b) including a solid–solid transition reaction between 2-line ferrihydrite and goethite at pH = 14.0. In both panels, the dashed and dotted lines correspond to the simulation results, including a low (*k*_2l→gt_ = 4 · 10^–11^ mol m^–2^ s^–1^) and high (*k*_2l→gt_ = 4 · 10^–10^ mol m^–2^ s^–1^) rate of solid–solid transformation.

In contrast to its precipitation velocity, the rate of 2-line ferrihydrite dissolution is correlated to the pH and proportional to the cube of the phase mass. The rate of goethite precipitation, on the other hand, is sensitive to the aqueous concentration of [Fe(OH)_4_^–^]^4^ and decreases exponentially with pH (Figure 4). For all simulations performed, the best fits were achieved, excluding a solid–solid transformation (*k*_2l→gt_ = 0 mol m^–2^ s^–1^). As displayed in Figure 5, the additional consideration of an aggregation-based transformation between 2-line ferrihydrite and goethite predicts aqueous Fe(OH)_4_^–^ concentrations within the confidence interval of concentration profiles predicted in its absence (Figure 5a). High solid–solid transformation rate constants in the orders of 10^–10^ mol m^–2^ s^–1^ result in a marginally more accurate prediction of the solid fraction at low equilibration times, but grossly overpredict the rate of 2-line ferrihydrite conversion in the long term (Figure 5b). Moreover, the designated initial precipitation rate of the more soluble, amorphous precursor 2-line ferrihydrite is much larger than the rate of goethite precipitation . The emerging transformation mechanism, consisting of the rapid precipitation of 2-line ferrihydrite, followed by its dissolution and the slow precipitation of goethite from solution, is a multistep process of which each step is in complete agreement with the principles of classical nucleation theory^35^ as well as Stranski’s rule^30^ and the Ostwald step rule.^31^

Even though the surface rate constant of goethite precipitation is 10^23^–10^25^ times higher than the rate of 2-line ferrihydrite dissolution, the effective reaction rates evaluated at the measured aqueous *n*_Fe(OH)_4_^–^_^4^ and solid *n*_2l_^3^ over time are of comparable magnitude (Supporting Information, Figure S1c). At low equilibration times, the rapid decrease in *n*_Fe(OH)_4_^–^_ and the corresponding reduction in the degree of supersaturation with respect to goethite appear to attenuate the high precipitation rate constant. Likewise, the specific surface area of goethite reduces to about 10% of its initial value within the first 3 h of the experiment (Supporting Information, Figure S1d). Over the entire timespan investigated, the rate of 2-line ferrihydrite dissolution remains below the rate of goethite precipitation, as evidenced by the strictly monotonic decrease in the aqueous Fe(OH)_4_^–^ concentration measured by ICP-OES, and can thus be considered the rate-limiting step of the transformation mechanism. It can furthermore be shown that, for a specific dependence of *dn*_*j,t*_/*dt* on the specific surface area *A*_*s,j,t*_, the evolution of *n*_*j,t*_ follows the progression
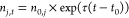
13where τ = *M*_*w,j*_*A*_*s,j*,0_*k*_*j*_*n*_*j*,0_^3^ in s^–1^. For a more detailed account of the derivation of eq 13, the reader is referred to the Supporting Information. At constant pH and sample mass, the rate of 2-line ferrihydrite dissolution is first order with respect to *n*_*j,t*_ and the time constant of dissolution τ depends entirely on the initial sample surface area.

### The Precipitation of Fe(III) at Neutral to Alkaline pH

As elucidated in the previous section, the rate of goethite precipitation is highly sensitive to the aqueous concentration of Fe(OH)_4_^–^. This association between precipitation velocity and the predominant aqueous Fe(III) hydrolysis product was documented in previous studies. Pham et al.^29^ established an analogous relationship between the rate of Fe(III) precipitation and the concentration of Fe(OH)_3_(aq) at pH = 6.0–9.5, i.e., across the predominance interval of Fe(OH)_3_(aq), and obtained an intrinsic precipitation rate constant of *k*_Fe(OH)_3_(aq)_ = 2.0 · 10^7^ L mol^–1^ s^–1^. Even though Fe(III) is known to precipitate as mixtures 2-line ferrihydrite, goethite, and hematite at circumneutral pH,^17,19^*k*_Fe(OH)_3_(aq)_ can be compared to the transformation rates quoted in this study under the assumption that the rate of 2-line ferrihydrite dissolution is rate-limiting. The observed relationship (Figure 4) between the rate of goethite precipitation and the concentration of Fe(OH)_4_^–^ at pH = 13.0–14.0, i.e., across the predominance interval of Fe(OH)_4_^–^, suggests that the precipitation rate of Fe(III) follows the solubility limit of the solid Fe(III) phase stabilized. Moreover, differences in the precipitation mechanism at acidic, neutral, and alkaline pH appear to be a consequence of the different Fe(III) coordination environments. The partial equilibrium model developed in this paper can hence be extended to any pH, provided the thermodynamic speciation solver includes the respective predominant aqueous Fe(III) species correlated with the precipitation velocity. At acidic pH beyond the range considered in Pham et al.^29^ and this study, Fe(III) is predominantly coordinated as the trivalent cation Fe^3+^ (pH = 0.0–2.8), FeOH^2+^ (pH = 2.8–3.9), and Fe(OH)_2_^+^ (pH = 3.9–6.4).^32^ In addition to the predominant Fe(III) hydrolysis products, the formation of polynuclear ferric species including Fe_2_(OH)_2_^4+^ and Fe_3_(OH)_4_^5+^ makes up a significant portion of the total aqueous molar fraction at highly alkaline pH < 2.^32,36^ According to the pH-dependent speciation, the total rate constant of Fe(III) precipitation
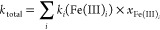
14will be a combination of the individual rate constants of all species contributing to the solubility limit *k*_*i*_(Fe(III)_*i*_) and their aqueous molar fractions *x*_Fe(III)_*i*__. Depending on the crystallization conditions and the presence of other complexing ions, the overall precipitation rate may further be influenced by the formation of aqueous complexes including iron chlorides and silicates^25,37,38^ as well as other transformation pathways including the recrystallization of solid Fe(III) phases other than the here investigated 2-line ferrihydrite and goethite.^17^ Nevertheless, the total rate of Fe(III) precipitation as formulated in this section holds true, provided that (i) 2-line ferrihydrite is the solubility limiting Fe(III) phase and (ii) Fe(OH)_3_(aq) and Fe(OH)_4_^–^ are the predominant aqueous hydrolysis complexes at pH = 6.0– 9.5 and pH = 9.5–14.0, respectively.

**Figure 6:**
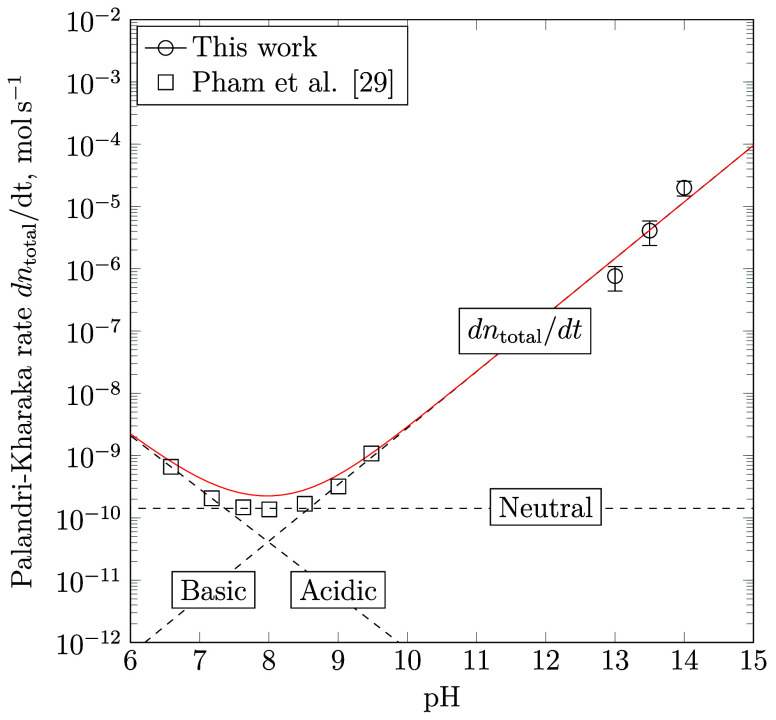
Estimated Palandri–Kharaka-type precipitation rate of Fe(III) d*n*_total_/d*t* in mol s^–1^ at neutral to alkaline pH (solid line), together with the individual mechanistic acidic, neutral and basic contributions to d*n*_total_/d*t* (dashed lines), obtained via linear regression of the experimentally measured precipitation rates (symbols) at pH = 6.5–7.1, 7.6–8.5 and 9.4–14.0, respectively.

To enable better comparison between the rates goethite precipitation presented in this study and those computed by Pham et al.,^29^ various *k*_*j*_ values have been normalized to a surface area of 1 m^2^, multiplied by the solubility limit of 2-line ferrihydrite at pH = 6.0–14.0^32^ and reformulated in terms of the H^+^ activity instead of the pH. The here presented novel empirical fitting results and the previously obtained intrinsic rate of Fe(III) precipitation are thereby expressed in terms of the Palandri and Kharaka^39^ kinetic formalism, where the overall rate of transformation,
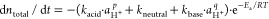
15consists of 3 individual contributions at acidic, neutral, and alkaline pH. In eq 15, *E*_a_ stands for the activation energy in *J* mol^–1^, *R* denotes the ideal gas constant in J mol^–1^ K^–1^, and all other parameters have their usual meanings. Figure 6 displays the fitted overall rate of Fe(III) precipitation, d*n*_total_/d*t* in mol s^–1^. The kinetic parameters of each contribution to the overall rate of precipitation are obtained by segregating the experimental data into an acidic (pH = 6.5–7.1), near-neutral (pH = 7.6–8.5) and basic (pH = 9.4–14.0) region and then performing a piecewise linear regression on each pH interval.

It is found that the rate constants of Fe(III) precipitation decrease from log_10_* k* = −1.49 at acidic to log_10_* k* = −7.80 at neutral and log_10_* k* = −15.59 at alkaline pH. The overall transformation rate is slightly weaker correlated with *a*_H^+^_ at acidic pH (*p* = −0.857) as opposed to the basic regime (*q* = 0.908), though various acidic rate parameters may be subject to further revision due to the lack of data points at pH < 6.5. Irrespective of the pH, the activation energy is estimated to be *E*_a_ = 11.7 kJ mol^–1^. It is also remarkable that the precipitation velocity at circumneutral pH is close to the dissolution rate of goethite (log_10_* k* = −7.94), as determined by Palandri and Kharaka.^39^ These findings underline the crucial role of the pH-dependent speciation of iron as a rate-limiting factor in the formation of thermodynamically stable, crystalline Fe(III) end members.

### Environmental Implications

The analysis carried out in this paper demonstrates that the rate of crystalline Fe(III) (hydr)oxide formation is highly sensitive to the coordination environment of Fe(III) in the aqueous phase. At highly alkaline pH > 13, i.e., across the thermodynamic stability domain of Fe(OH)_4_^–^, precipitation occurs rapidly in the orders of 10^–7^ (pH = 13) to 10^–4^ (pH = 14) mol s^–1^. At neutral (pH = 7) to mildly alkaline (pH = 10) conditions, i.e., characteristic to uranium mine tailings,^18^ Fe(III) is predominantly coordinated as Fe(OH)_3_(aq). This shift in the coordination environment coincides with a reduction in the incident rate of Fe(III) (hydr)oxide formation by 2–5 orders of magnitude. It is further highlighted that the accelerated stabilization of crystalline Fe(III) end members proceeds via and is rate-limited by the dissolution of the amorphous iron hydroxide 2-line ferrihydrite. These observations have severe implications for the sequestration of contaminants including heavy metals from groundwater streams^3^ and hazardous radionuclides in nuclear processing facilities.^5^ As 2-line ferrihydrite has a strong affinity for adsorbing and coprecipitating contaminants such as heavy metals, it is expected that the accelerated rate of dissolution (Figure 4) and the strong dependency of the overall rate of crystalline Fe(III) precipitation on the H^+^ activity (Figure 6) enhance the mobility and bioavailability of elements of concern at alkaline conditions. A more thorough understanding of their sequestration must thus be investigated under consideration of all mechanistic steps that govern the formation of amorphous and crystalline iron (hydr)oxides, including the pH-dependent speciation of Fe(III) in the Fe–H_2_O system.

## Methods

The formation of
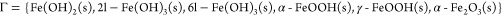
from and in the presence of
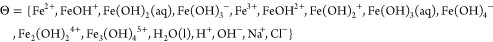
can be described by a sequence of partial equilibrium steps. It is assumed that one or more Γ is out of equilibrium with the remaining species and all Θ are in equilibrium with one another. Depending on the time-dependent evolution of the aqueous species, the rate of mineral dissolution and growth  is formulated as a function of their bulk thermodynamic quantities, the phase saturation index Ω_*j,t*_ and the activity of various Θ the formation of *j* ∈ Γ is sensitive to. Analogous to the seeded growth modeling of other minerals including portlandite^40^ and calcite,^41^ changes to the particle geometry are considered by correcting for changes in the particle surface area upon each time step of the simulation.

### Gibbs Free Energy Minimization

Mineralogical phase equilibria and bulk compositions were determined using the Reaktoro framework,^42^ utilizing a custom thermodynamic database of Fe(II) and Fe(III) complexes and solid phases previously published in Furcas et al.,^32^ accompanied by selected auxiliary species taken from Grenthe et al.^43^ and Hummel et al.^44^ The mineral–water interaction of Γ can be described as
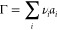
16where ν_*i*_ and *a*_*i*_ are the stoichiometric coefficient and activity of species *i* ∈ Θ, respectively. With *n*^(*x*)^ being the bulk composition at equilibrium and *n*^(*b*)^ representing the initial number of atoms present, the system’s total Gibbs free energy
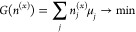
17is minimized subject to the molar balance
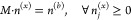
18where *M* is the component-wise matrix of atomic balance coefficients.

### Calculation of the Activity Coefficients

We denote the chemical potential of each constituent of the aqueous electrolyte solution *i* as
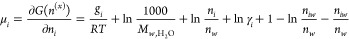
19where *g*_*i*_ is the partial molar Gibbs free energy in J mol^–1^; *M*_*w*,H_2_O_ = 18.0153 g mol^–1^ is the molecular weight of liquid water; and *n*_*w*_, *n*_*i*_, and *n*_*iw*_ represent the total number of moles of the aqueous phase, of constituent *i* and of the water solvent *iw*.^45,46^ For each species, the activity coefficient γ_*i*_ is computed according to the extended Debye–Hückel equation in Truesdell–Jones form
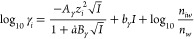
20where *b*_γ_ ∼ 0.098 for NaOH,
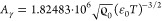
21
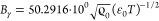
22and the effective ionic strength
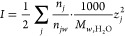
23is computed from the charge of each species *z*_*j*_ and their respective molarity, relative to 1 kg of water.^47^ Considering the density ϱ_0_ and dielectric constant ε_0_ of pure water at *T_r_* = 298.15 K and *P*_*r*_ = 1 bar, the Debye–Hückel limiting law parameters are *A*_γ_ ∼ 0.5114 and *B*_γ_ ∼ 0.3288.

### Implementation of Mineral–Water Reaction Kinetics

Dissolution and growth kinetics are incorporated in the minimization routine via a series of partial, rather than complete equilibria, as described by Kulik and Thien.^48^ The number of solid species *n*_*j*_^(*x*)^ ∈ Γ is changed based on its saturation index Ω_*j*_.^49^ For
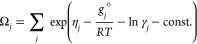
24where η_*j*_ is the dual-solution chemical potential, *g*_*j*_^°^ is the standard Gibbs free energy in J mol^–1^, and γ_*j*_ is the activity coefficient of phase *j*. The number of moles *n*_*j*_^(*x*)^ at time step *t* + Δ*t* may be computed as
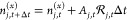
25for
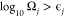
26and
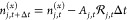
27for
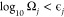
28where *A*_*j,t*_ is the total particle surface area, *R*_*j,t*_ is the rate of phase growth in mol m^–2^ s^–1^, and ϵ_*j*_ is the stability criterion for phase *j* at time *t*. These series simulate the stepwise precipitation from supersaturation (log_10_ Ω_*j*_ > 0) and dissolution in undersaturated conditions (log_10_ Ω_*j*_ < 0). The kinetic rate laws that govern changes to the bulk elemental composition of the chemical system due to the formation and dissolution of Γ may be written as
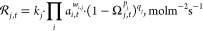
29∀ *i* ∈ Θ, ∀*j* ∈ Γ, ∀*t*, where *k*_*j*_ is the reaction rate constant and Ω_*j,t*_ denotes the dimensionless saturation index of species *j* at time *t*. Moreover, *a*_*i,t*_ represents the activity of species *i* at time *t* and *w*_*i,j*_, *p*_*j*_, and *q*_*j*_ are treated as empirical parameters. For a specific molar volume of *V*_*m,j*_ in m^3^ mol^–1^, the mean orthogonal velocity of surface propagation  is related to the rate of phase formation , according to
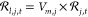
30In these Palandri–Kharaka-type reaction rate expressions,^39^ the saturation index is evaluated directly from the dual-solution chemical potential of phase *j*, as displayed in eq 24.

### Surface Area and Morphology Correction

**Figure 7:**
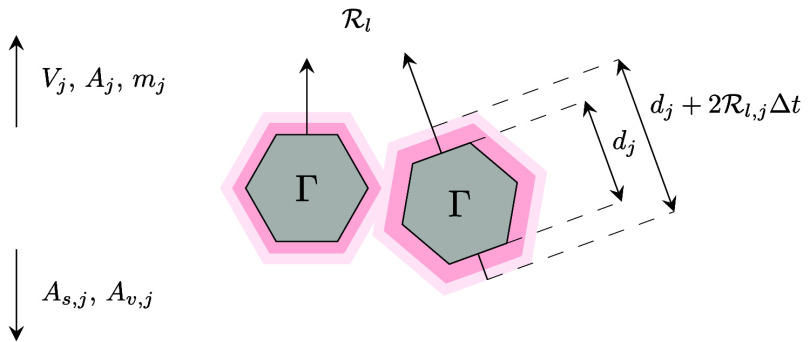
Schematic illustration of the growth process of mineral particles at the mean orthogonal velocity of surface propagation . The resultant increase of the particle diameter and thus particle volume *V*_*j*_, surface area *A*_*j*_, and mass *m*_*j*_ causes a reduction in specific surface area *A*_*s,j*_ and surface area per unit volume *A*_*v,j*_.

Changes to the mineral surface area *A*_*j*_ are incorporated into the molar balance of each phase *j* ∈ Γ by two different models part of the TKinMet library of the geochemical modeling package GEM-Selektor.^46,48^ Consider the growth of Γ, as schematically illustrated in Figure 7.

The increase of particle diameter *d*_*j*_ at time *t* to  at time *t* + Δ*t* at the mean orthogonal rate of surface propagation  increases the particle volume *V*_*j*_, surface area *A*_*j*_ and mass *m*_*j*_, while the specific surface area *A*_*s,j*_ = *A*_*j*_/*m*_*j*_ and the area per unit volume *A*_*v,j*_ = *A*_*j*_/*V*_*j*_ are expected to reduce. This reduction can be computed from the initial specific surface area A_*s,j*,0_ by the simple cubic root correction
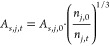
31where *n*_*j*,0_ and *n*_*j,t*_ represent the initial and final number of moles of phase *j*.^48,50^ The expected reduction to the surface area per unit volume can alternatively be described by
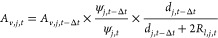
32where all parameters have their usual meaning. The shape factor ψ_*j*_ in eq 32 is equivalent to the sphericity coefficient, as described by Wadell^51^
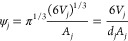
33where *V*_*j*_ and *A*_*j*_ are the mean particle volume and surface area and *d*_*j*_ = 6/(ψ_*j*_*A*_*j*_) is the estimated particle diameter. Further, changes in morphology upon dissolution and growth are accounted for by the shape factor function, expressed as a formal power series
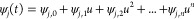
34of the phase saturation index *u* = log_10_ Ω_*j,t*_.^50^

## Data Availability

The data that support the findings of this study are available within the article and its Supporting Information.

## Supporting Information Available

Additional plots of the saturation indices of 2-line ferrihydrite and goethite, their dissolution and precipitation rates, and specific surface areas; derivation of the rate of 2-line ferrihydrite dissolution; and list of symbols and notations (PDF)

## Footnotes

1 The stoichiometric coefficients of all species in reaction 2 are ν_*i*_ = 1. They are omitted from the molar balance displayed in eq 4.

2 Apart from the stoichiometric coefficient of H_2_O(l) in the precipitation reaction of goethite, various other ν_*i*_ in reactions 6–8 equal to 1. They are omitted from the combined molar balance displayed in eq 12.
