# Supplementary material for: Speciation Controls the Kinetics of Iron Hydroxide Precipitation and Transformation at Alkaline pH
Source: Environ Sci Technol. 2024 Oct 23;58(44):19851–60. doi: 10.1021/acs.est.4c06818 (PMC11542892; doi:10.1021/acs.est.4c06818)
Supplement: Supplementary file 1 — es4c06818_si_001.pdf [file es4c06818_si_001.pdf]

Supporting Information for Publication

Speciation controls the kinetics of iron hydroxide precipitation and transformation

Fabio E. Furcas[1], Shishir Mundra[1], Barbara Lothenbach[2], Ueli M. Angst[1*]

[1]Institute for Building Materials, ETH Zürich, Laura-Hezner-Weg 7, 8093 Zürich, Switzerland, [*]email: uangst@ethz.ch
[2]Empa Concrete & Asphalt Laboratory, Ueberlandstrasse 129, 8600 Dübendorf, Switzerland

Synopsis: The transformation of 2-line ferrihydrite is rate-limited by its dissolution as aqueous Fe(III) across a broad range of natural and industrially relevant aqueous systems.

Keywords: precipitation, iron, kinetics, pH, partial equilibrium

Summary: 3 pages, 1 figure, 1 table

Additional plots

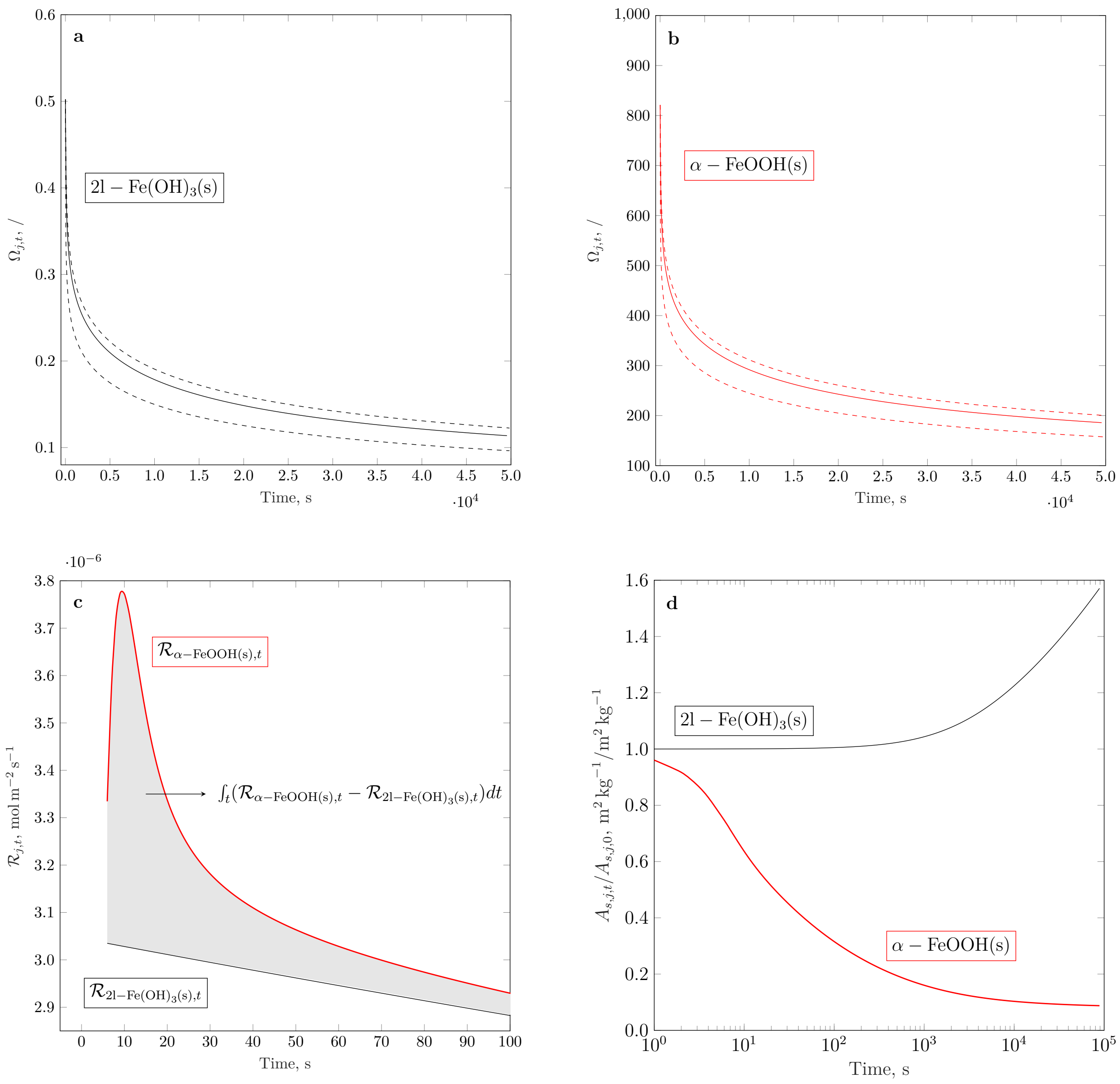

Figure S1: Saturation indices of species 2-line ferrihydrite (Figure S1a) and goethite (Figure S1b) at pH = 14.0 over time, together with the rates of 2-line ferrihydrite dissolution an goethite precipitation $\mathcal{R}_{j,t}$ in mol m^{-2} s^{-1} (Figure S1c) and their normalised specific surface areas $A_{s,j,t}/A_{s,j,0}$ in m^2 kg^{-1}/m^2 kg^{-1} (Figure S1d). Dashed lines represent the confidence interval of predicted saturation indices, corresponding to the upper and lower limit of reaction rate constants displayed in Figure 3.

Derivation of the rate of 2-line ferrihydrite dissolution

The molar rate of 2-line ferrihydrite dissolution

$$dn_{j,t}/dt = A_{j,t}\mathcal{R}_{j,t} = A_{s,j,t}M_{w,j}n_{j,t}k_jn_{j,t}^3 = A_{s,j,t}M_{w,j}k_jn_{j,t}^4 \qquad (1)$$

in $(\mathrm{m^2\,g^{-1}})\cdot(\mathrm{g\,mol^{-1}})\cdot(\mathrm{mol^{-2}\,m^{-2}\,s^{-1}})\cdot(\mathrm{mol^4})$ can be expressed in terms of the initial specific surface area, by using the cubic root correction, as displayed in Equation 31, or any other general correction formula

$$A_{s,j,t} = A_{s,j,0}\cdot\left(\frac{n_{j,0}}{n_{j,t}}\right)^{\alpha}. \qquad (\mathrm{m^2}) \quad (2)$$

Substituting $A_{s,j,t}$ in Equation 1 with Equation yields

$$dn_{j,t}/dt = A_{s,j,0}n_{j,0}^{\alpha}M_{w,j}k_jn_{j,t}^{4-\alpha}. \qquad (3)$$

in $(\mathrm{m^2\,g^{-1}})\cdot(\mathrm{mol^{\alpha}})\cdot(\mathrm{g\,mol^{-1}})\cdot(\mathrm{mol^{-2}\,m^{-2}\,s^{-1}})\cdot(\mathrm{mol^{4-\alpha}})$. Integrating from $n_{j,0}$ to $n_{j,t}$ and t_0 to t,

$$\int_{n_{j,0}}^{n_{j,t}} dn_{j,t}n_{j,t}^{\alpha-4} = A_{s,j,0}n_{j,0}^{\alpha}M_{w,j}k_j\int_{t_0}^{t} dt, \qquad (\mathrm{mol}) \quad (4)$$

the number of moles of ferrihydrite $n_{j,t}$ decay exponentially for $\alpha = 3$,

$$n_{j,t} = n_{j,0}\times\exp(A_{s,j,0}M_{w,j}k_jn_{0,j}^3(t-t_0)) = n_{j,0}\times\exp(\tau(t-t_0)), \qquad (\mathrm{mol}) \quad (5)$$

where $\tau = A_{s,j,0}M_{w,j}k_jn_{0,j}^3$ in $\mathrm{s^{-1}}$ is the time constant of dissolution. For $\alpha = 4$, $n_{j,t}$ reduces linearly according to

$$n_{j,t} = n_{j,0} + A_{s,j,0}M_{w,j}k_jn_{0,j}^4(t-t_0) = \tau(t-t_0), \qquad (\mathrm{mol}) \quad (6)$$

and for $\alpha \neq 3, 4$, the progression of $n_{j,t}$ is described by

$$n_{j,t} = \left(n_{j,0}^{\alpha-3} + (\alpha-3)A_{s,j,0}M_{w,j}k_jn_{0,j}^{\alpha}(t-t_0)\right)^{1/(\alpha-3)}. \qquad (\mathrm{mol}) \quad (7)$$

Note that τ in Equations 6 and , τ is not a real time constant and has units of $\mathrm{mol}^{\alpha-3}\mathrm{s}^{-1}$.

List of symbols and notations

Table S1: List of symbols and notations used in this paper. In addition to the parameter-specific subscripts listed in this table, indices i, j refer to the chemical species Γ, Θ and index t denotes time.

Symbol	Description
A	Particle surface area, $\mathrm{m^2}$
A_s	Specific surface area (SSA), $\mathrm{m^2\,kg^{-1}}$
A_v	Surface area per unit volume, $\mathrm{m^2\,m^{-3}}$
a	Chemical activity, /
d	Particle diameter, m
E_a	Activation energy, $\mathrm{J\,mol^{-1}}$
G	Total Gibbs free energy, J
g	Partial molar Gibbs free energy, $\mathrm{J\,mol^{-1}}$
g°	Standard molar Gibbs free energy, $\mathrm{J\,mol^{-1}}$
γ	Activity coefficient, /
ν	Dual-solution chemical potential, /
ϵ	Phase stability criterion, /
ε	Dielectric constant, /
I	Effective ionic strength, $\mathrm{mol\,kg^{-1}}$
k	Reaction rate constant, $\mathrm{mol\,m^{-2}\,s^{-1}}$
M_w	Molecular weight, $\mathrm{g\,mol^{-1}}$
m	Particle mass, kg
μ	Chemical potential, /
n	Number of moles, mol
ν	Stoichiometric coefficient, /
Ω	Saturation index, /
P	Pressure, bar
p	Empirical parameter, /
q	Empirical parameter, /
ψ	Wadell sphericity[38] , /
$\mathcal{R}$	Rate of phase formation, $\mathrm{mol\,m^{-2}\,s^{-1}}$
$\mathcal{R}_l$	Mean orthogonal rate of surface propagation, $\mathrm{m\,s^{-1}}$
ϱ	Density, $\mathrm{kg\,m^{-3}}$
T	Temperature, K
u	Decadic logarithm of the phase saturation index, /
V	Particle volume, $\mathrm{m^3}$
V_m	Specific molar volume, $\mathrm{m^3\,mol^{-1}}$
w	Reaction order term, /
z	Formal charge, /
[ ]	Concentration, $\mathrm{mol\,L^{-1}}$
